# Role of Single Nucleotide Polymorphism-Related Genes in Tumour Immune Cell Infiltration and Prognosis of Cutaneous Melanoma

**DOI:** 10.1155/2023/3754094

**Published:** 2023-05-09

**Authors:** Baihe Wang, Fanxiao Liu, Yuanyuan Li, Nan Chen

**Affiliations:** Department of Dermatology, Shandong Provincial Hospital Affiliated to Shandong First Medical University, Jinan, Shandong 250021, China

## Abstract

**Background:**

Advances in cancer research have allowed for early diagnosis and improved treatment of cutaneous melanoma (CM). However, its invasiveness and recurrent metastasis, along with rising resistance to newer therapies, have lent urgency to the search for novel biomarkers and the underlying molecular mechanisms of CM.

**Methods:**

Single nucleotide polymorphism- (SNP-) related genes were obtained from the sequencing data of 428 CM samples in The Cancer Genome Atlas. Functional enrichment of these genes was analysed in clusterProfiler. Additionally, a protein-protein interaction (PPI) network was constructed with the Search Tool for the Retrieval of Interacting Gene (STRING) database. Gene Expression Profiling Interactive Analysis (GEPIA) was used to identify the expression and prognostic value of mutated genes. Finally, the Tumour Immune Estimation Resource (TIMER) analysed the relationship between gene expression and immune cell infiltration.

**Results:**

We constructed a PPI network from the top 60 SNP-related genes. Mutated genes were mainly involved in calcium and oxytocin signalling pathways, as well as circadian entrainment. In addition, three SNP-related genes, *BRAF*, *FLG*, and *SORL1*, were significantly associated with patient prognosis. *BRAF* and *SORL1* were positively associated with infiltration abundance of B cells, CD8+ T cells, CD4+ T cells, neutrophils, and dendritic cells, whereas *FLG* expression was negatively associated. Furthermore, higher immune cell infiltration was positively correlated with good prognosis.

**Conclusions:**

Our study provides vital bioinformatic data and a relevant theoretical basis to further explore the molecular pathogenesis of CM and improve patient prognosis.

## 1. Introduction

Melanoma affects over 300,000 people worldwide annually [1], while survival in cutaneous melanoma (CM) has greatly increased with advancements in treatment, including the development of targeted therapy and immunotherapy [2]. However, a subset of patients became resistant. Identifying oncogenic biomarkers can help guide treatment decisions and elucidate the determinants of responses to immune and targeted therapies.

Single nucleotide polymorphisms (SNPs) can arise through transformation, transversion, deletion, or insertion. Because SNPs frequently occur throughout the genome, they provide an opportunity to identify mutations associated with a wide range of diseases, including cancers [3]. Increasing evidence suggests that susceptibility to malignant CM is associated with SNPs [4]. *GLI-1* polymorphisms in the hedgehog pathway are risk factors for melanoma susceptibility and can be used as prognostic biomarkers [5]. Furthermore, the *IRF4* SNP (rs12203592) and the *MTAP* (rs869330) variants are both associated with melanoma-specific survival [6]. Given their likelihood of influencing prognosis, an analysis of relevant SNPs could help identify new prognostic biomarkers for patients with CM.

The tumour microenvironment influences gene expression in tumour tissues and consequently has a strong effect on clinical outcomes [7–10]. Furthermore, immune cells in the microenvironment can be used for the prognostic assessment of multiple tumours, such as glioblastoma, breast cancer, and melanoma [11–13]. Notably, CM has a highly activated tumour microenvironment, with most immune system components involved in the cancer's initiation and progression [14]. The degree of immune cell infiltration significantly affects melanoma prognosis [15].

In this study, we aimed to identify novel immune-related prognostic biomarkers for CM and explore their underlying molecular mechanisms. To investigate the biological significance of SNPs in CM prognosis, we obtained SNP-related genes from The Cancer Genome Atlas (TCGA) and performed bioinformatic analysis. The results provide a theoretical basis for researchers to develop personalised treatment methods for patients with CM.

## 2. Materials and Methods

### 2.1. Data Acquisition

As the largest cancer gene database available, TCGA (https://portal.gdc.cancer.gov/) houses data on cancer gene expression, miRNA expression, copy number variants, methylation, and SNPs [16]. However, original SNP data are not available to the public. Instead, relevant SNP and raw mRNA expression data of patients with CM were downloaded. Mutation data were analysed with the R Bioconductor package, “Maftools” [17].

### 2.2. Functional Analysis

The R package “clusterProfiler” was used for Gene Ontology (GO) and Kyoto Encyclopedia of Genes and Genomes (KEGG) analyses [18]. The cut-off for significant enrichment was *P* < 0.05. Mutated genes were screened for enrichment in molecular functions, biological processes, cellular components, and biological pathways.

### 2.3. Protein-Protein Interaction (PPI) Network Construction

The Search Tool for the Retrieval of Interacting Gene (STRING) database predicts physical and functional interactions between proteins [19]. Interactions, nodes, and subnetworks of the top 60 mutated genes were analysed by Cytoscape [20], a program that imports STRING networks but also integrates data from associated databases.

### 2.4. Survival Analyses and Identification of Potential Prognostic Biomarkers

Patient survival was assessed using the Kaplan-Meier plots of results from gene arrays, RNA sequencing, and next-generation sequencing. These data were also used to screen for potential prognostic biomarkers by assessing the effect of mutant gene expression on patient prognosis with the Gene Expression Profiling Interactive Analysis (http://gepia.cancer-pku.cn) [21].

### 2.5. TIMER and Multivariate Cox Regression

The Tumour Immune Estimation Resource (TIMER) (http://timer.cistrome.org/) can use RNA sequencing data to detect associations between mutated genes and immune cell infiltration [22]. In this study, TIMER was used to perform a multivariate Cox regression on cells involved in immune cell infiltration, including CD4+ T cells, CD8+ T cells, B cells, macrophages, and neutrophils. Hazard ratios and 95% confidence intervals were also calculated. CIBERSORT in R was then used to identify immune cell subtypes and determine the relationship between those cells and risk scores [23]. In this study, we assessed the relationship between *BRAF*, filaggrin (*FLG*), and sortilin-related receptor 1 (*SORL1*) expressions and immune cell infiltration in patients with CM.

## 3. Results

### 3.1. Mutation Profiles in CM Samples

**Figure 1:**
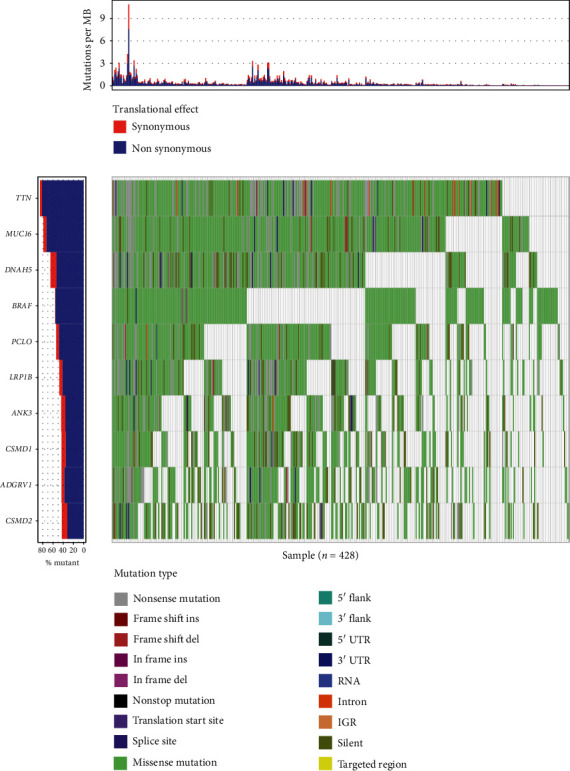
The landscape of mutation data of TCGA CM dataset. Mutation information of each gene in each sample is displayed in the waterfall plot, where different colors with specific annotations at the right bottom mean the various mutation types. The barplot above the legend exhibits the number of mutation burden.

We downloaded level-three transcriptome data for all available CM samples (*n* = 428). Analysis in Maftools revealed that the top 10 mutated genes in CM samples were *TTN*, *MUC16*, *DNAH5*, *BRAF*, *PCLO*, *LRP1B*, *ANK3*, *CSMD1*, *ADGRV1*, and *CSMD2* (Figure 1).

### 3.2. Functional Enrichment Analysis

**Figure 2:**
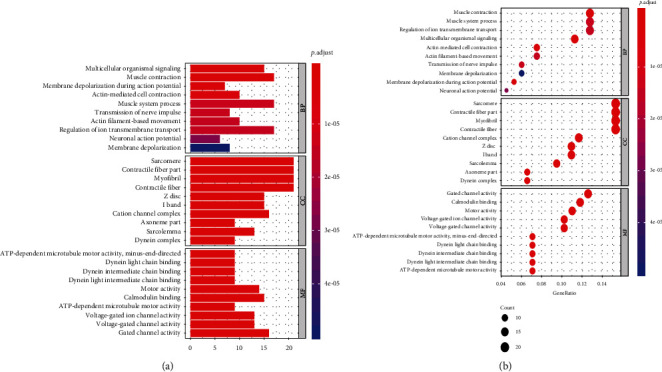
GO functional enrichment analysis. The dot indicates the gene cluster. The redder the color of the dots, the more significant the GO term. MF: molecular function; CC: cellular component; BP: biological process.

**Figure 3:**
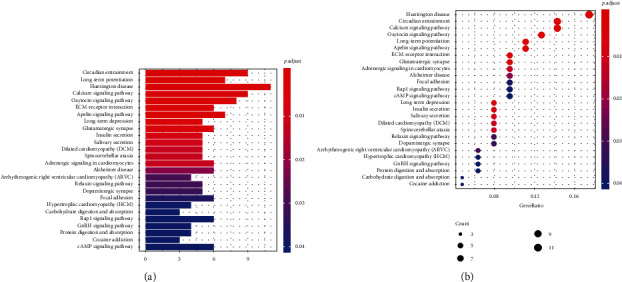
KEGG analysis showed that mutated genes were mainly enriched in the calcium and oxytocin signalling pathway and circadian entrainment. The redder the color of the dots, the more significant the KEGG term.

The results of GO analysis on mutated genes revealed enrichment in “multicellular organismal signalling”, “muscle system process”, and “regulation of ion transmembrane transport” terms under the biological process category. In the cellular component category, terms “sarcomere”, “contractile fibre part”, and “myofibril” were enriched. Lastly, in the molecular function category, the terms “gated channel activity”, “calmodulin binding”, and “motor activity” were enriched (Figures 2(a) and 2(b)). Results from the KEGG analysis showed that mutated genes were enriched in the calcium and oxytocin signalling pathways, as well as in circadian entrainment pathways (Figures 3(a) and 3(b)).

### 3.3. PPI Network and Correlation Analysis of Mutated Genes

**Figure 4:**
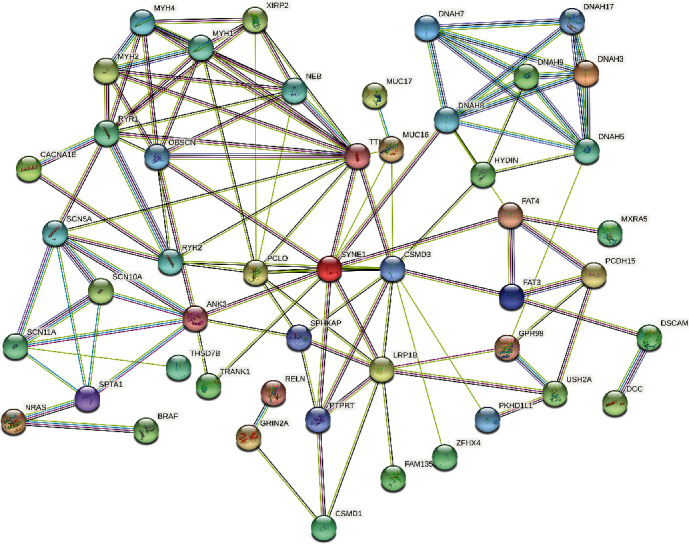
Protein-protein interaction network. The spheres represent proteins, and the lines represent protein interactions. The stronger the interaction between two proteins, the thicker the connection line, and the lines of different colors indicate different interactions.

We explored the correlation between mutated genes using the STRING database and then constructed a PPI network (Figure 4). We found correlations between mutations and expression of the top 60 genes.

### 3.4. Survival Analysis of Mutated Genes and Screening of Prognostic Biomarkers

**Figure 5:**
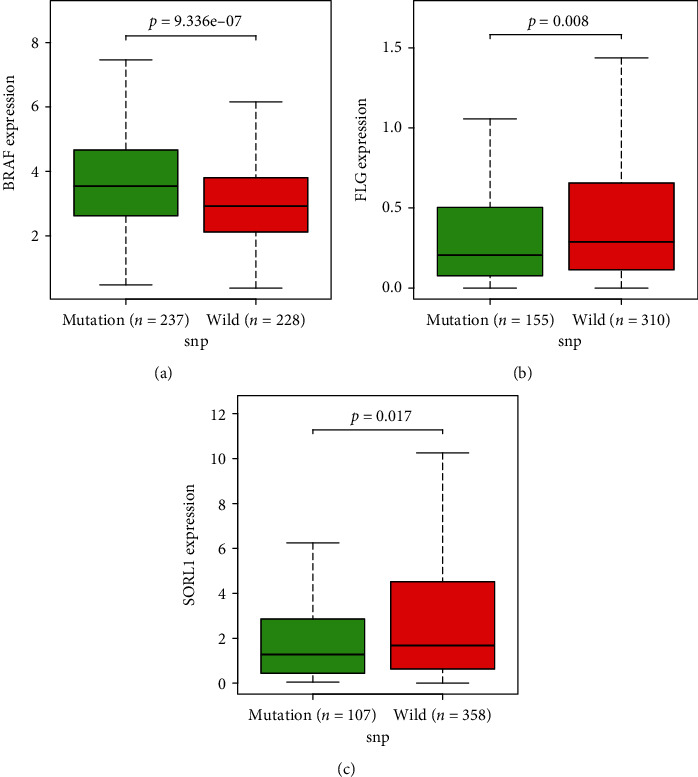
Box plots show the expression profiles of *BRAF* (a), *FLG* (b), and *SORL1* (c) in gene mutation patients with CM compared to those in wild samples. Patients are stratified into mutation and wild groups. *P* < 0.05 is considered statistically significant.

**Figure 6:**
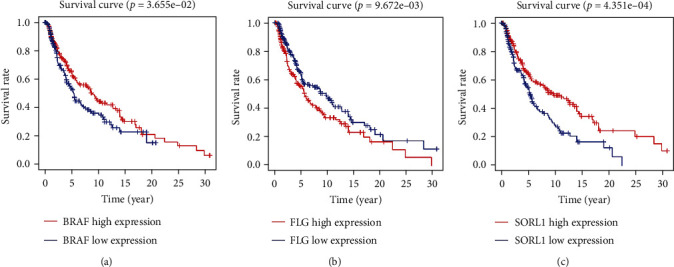
Survival curves of three SNP-related genes. *BRAF* (a) and *SORL1* (c) are positively associated with survival rate, while *FLG* (b) is negatively associated with survival rate.

We then explored the relationship between the mutated genes, their expression, and prognosis. Using *P* < 0.05 as the significance level, three mutant genes that significantly correlated with prognosis were identified: *BRAF*, *FLG*, and *SORL1*. The expression of *BRAF* and *SORL1* in the tumour samples was increased, while the expression of *FLG* was decreased (Figures 5(a)–5(c)). Based on the data, patients were divided into high- and low-expression groups according to the median expression values. The Kaplan-Meier plot results showed that increased expression of *BRAF* and *SORL1* was associated with a better prognosis (*P* = 3.655*e* − 02 and *P* = 4.351*e* − 04, respectively). In contrast, increased *FLG* expression resulted in poor prognosis (*P* = 9.672*e* − 03) (Figures 6(a)–6(c)). Moreover, both *in vitro* and *in vivo* experiments should be conducted in future studies.

### 3.5. Correlation between Mutated Genes and Immune Cell Infiltration

**Figure 7:**
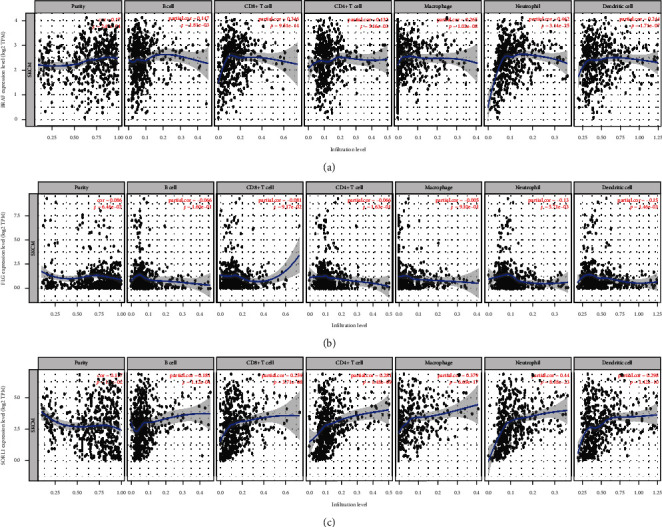
Correlation of three mutant genes with immune infiltration level in CM. The expression of *BRAF* (a) and *SORL1* (c) is positively associated with the infiltration abundance of B cells, CD8+ T cells, CD4+ T cells, macrophages, neutrophils, and dendritic cells, while *FLG* (b) is negative with the immune cell infiltration.

**Figure 8:**
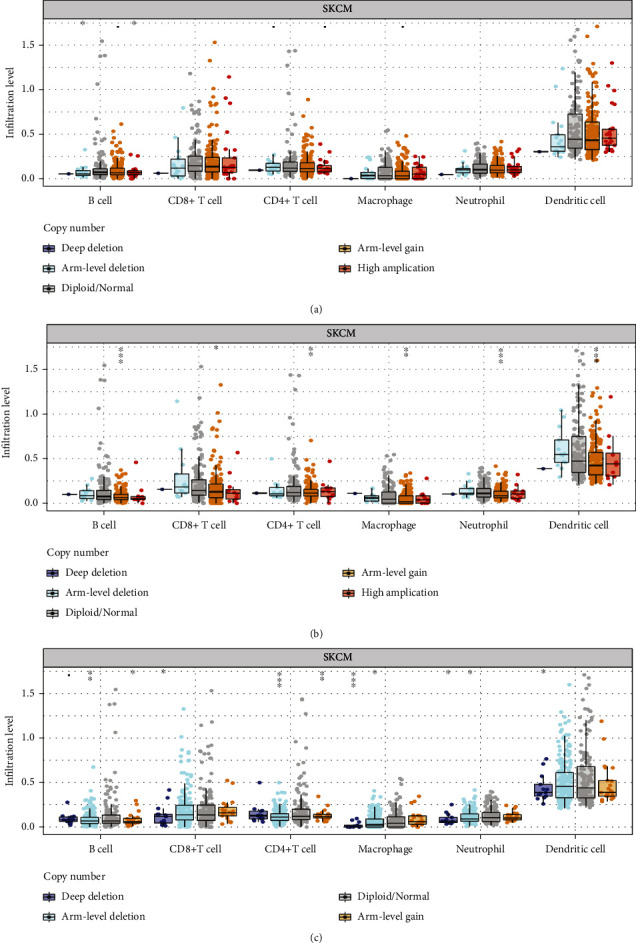
Associations between mutation types with immune cell infiltration of three mutant genes ((a–c) *BRAF*, *FLG*, and *SORL1*): mutation types (deep deletion, arm-level deletion, arm-level gain, and high amplification) of mutant-associated genes exhibit low level of immune cell infiltration compared with diploid/normal.

**Figure 9:**
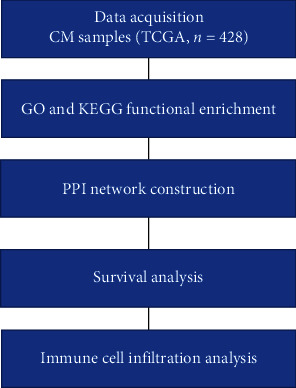
A flow chart of our study.

In patients with CM, the results showed that *BRAF* expression was highly correlated with CD8+ T cell (*r* = 0.346, *P* = 9.51*e* − 14) and neutrophil (*r* = 0.462, *P* = 3.14*e* − 25) infiltration (Figure 7(a)). *FLG* expression was highly correlated with dendritic cells (*r* = −0.15, *P* = 1.46*e* − 03) and neutrophil (*r* = −0.13, *P* = 5.71*e* − 03) infiltration (Figure 7(b)). *SORL1* expression correlated with macrophages (*r* = 0.379, *P* = 6.69*e* − 17) and neutrophil (*r* = 0.44, *P* = 8.18*e* − 23) infiltration (Figure 7(c)). Furthermore, we found that the infiltration of CD8+ T cells, dendritic cells, CD4+ T cells, neutrophils, and macrophages depended on gene mutation types in CM (Figures 8(a)–8(c)). For a flow chart of all steps in our study, see Figure 9.

## 4. Discussion

One of the most aggressive skin cancers, CM accounts for approximately 90% of global deaths attributed to this malignancy [24], while new treatments and early diagnosis have decreased mortality. However, 15%–20% of melanoma tumours do not respond to targeted therapies [25]. Moreover, treatment with programmed cell death protein 1 or cytotoxic T lymphocyte-associated antigen 4 antibodies does not benefit 40%–60% of patients [26]. Therefore, novel drug targets and their underlying mechanisms should be discovered to improve the assessment of malignancy and prognosis.

Our study identified three core mutated genes in patients with CM: *BRAF*, *FLG*, and *SORL1*. These genes were significantly associated with the prognosis of patients with CM. We thus consider mutant SNPs in these genes to be potentially carcinogenic markers that should benefit the early diagnosis and the design of individualised targeted therapy for CM.

A member of the RAF kinase family, *BRAF* mutations are common in many cancer types, including melanomas (60% occurrence), thyroid cancers (60%), colorectal cancers (15%), and non-small-cell lung cancers (5%–8%) [27]. *BRAF* inhibitors interfere with the mitogen-activated protein kinase (MAPK) signalling pathway that regulates melanoma proliferation and survival [28]. The MAPK pathway is also involved in T cell receptor signalling. Thus, *BRAF* interference exerts anticancer effects on the tumour microenvironment. Specifically, it increases intratumoural T cell infiltration and activity, decreases immunosuppressive cytokine levels, enhances melanoma-differentiation antigen expression, and introduces tumour antigens by regulating HLA-1 levels [29]. Through promoting tumour recognition by the immune system, these processes enhance antitumour T cell responses.

*FLG* is a pivotal structural protein of the stratum corneum; it encodes natural moisturising factors that are important to skin barrier function [30]. Thus, *FLG* mutation carriers may develop malignant melanoma [31]. Moreover, *FLG* is involved in allograft rejection and tumour necrosis factor-alpha signalling which is the primary pathway involved in interferon gamma response [32].

Lastly, *SORL1* is involved in DNA repair and oxidative phosphorylation. *SORL1* coprecipitates with endosomal receptor *HER2* in cancer cells and recycles it back to the plasma membrane, thus regulating its subcellular distribution [33]. *SORL1* is also involved in retromer-related endosomal trafficking and is a risk gene for Alzheimer's disease [34].

Functional analyses were performed to further investigate the molecular mechanisms of these mutant genes in CM progression. The results of GO analysis indicated enrichment in multicellular organismal signalling, muscle system processes, ion transmembrane transport, and gated channel activity. Additionally, KEGG analysis showed enrichment in the calcium and oxytocin signalling pathways, as well as in circadian entrainment.

We also found that *BRAF* and *SORL1* expressions were positively associated with immune cell (B cells, CD8+ T cells, CD4+ T cells, macrophages, neutrophils, and dendritic cells) infiltration in patients with CM. Additionally, their expression was positively correlated with improved prognosis, whereas mutated *FLG* expression was negatively correlated. Thus, higher levels of immune cell infiltration appear to be associated with better survival outcomes. To some extent, these patterns are consistent with previous findings suggesting that highly immune-infiltrated lung cancer evaded immune attacks by inhibiting new antigen expression [35]. Furthermore, a strong body of evidence indicates that lymphocyte infiltration in the tumour microenvironment is correlated with immunotherapeutic benefits [36–39]. Taken together, the data imply that the prognostic value of *BRAF*, *FLG*, and *SORL1* is associated with immune cell infiltration. An in-depth analysis of any unique properties of immune cell infiltration may aid in the development of cancer immunotherapy targets.

It is reported that an imbalance in the immune cell component ratio is highly correlated with poor prognosis and low survival in patients with cancer [40, 41]. In our study, we found that the differential expression of immune cell infiltration levels of three mutant genes depends on gene mutation types.

Our study had some limitations. We did not perform *in vivo* experiments to confirm the potential relationship between mutant genes, prognosis, and immune cell infiltration. Future studies that consider gene-gene and gene-environment interactions are warranted to better clarify the molecular mechanisms of CM.

## 5. Conclusions

In summary, we identify novel biomarkers by elucidating the role of SNP-related genes in tumour immune infiltration and the prognosis of patients with CM. Our study provides new perspectives for the identification of prognostic indicators and offers an opportunity to optimise CM treatment.

## Data Availability

The analyzed datasets generated during the study are available from the corresponding author on reasonable request.
